# Association of HLA-A and Non-Classical HLA Class I Alleles

**DOI:** 10.1371/journal.pone.0163570

**Published:** 2016-10-04

**Authors:** Federico Carlini, Virginia Ferreira, Stéphane Buhler, Audrey Tous, Jean-François Eliaou, Céline René, Jacques Chiaroni, Christophe Picard, Julie Di Cristofaro

**Affiliations:** 1 Etablissement Français du Sang Alpes Méditerranée, Marseille, France; 2 Aix Marseille Univ, CNRS, EFS, ADES, "Biologie des Groupes Sanguins", Marseille, France; 3 Laboratory of Anthropology, Genetics and Peopling history (AGP), Department of Genetics and Evolution–Anthropology Unit, University of Geneva, Geneva, Switzerland; 4 Transplantation Immunology Unit and National Reference Laboratory for Histocompatibility, Department of Genetic and Laboratory Medicine, Geneva University Hospitals, Geneva, Switzerland; 5 Department of Immunology, CHRU de Montpellier, University Hospital Saint-Eloi, Montpellier, France; 6 Faculté de Médecine, University of Montpellier 1, Montpellier, France; University of East London, UNITED KINGDOM

## Abstract

The HLA-A locus is surrounded by HLA class Ib genes: HLA-E, HLA-H, HLA-G and HLA-F. HLA class Ib molecules are involved in immuno-modulation with a central role for HLA-G and HLA-E, an emerging role for HLA-F and a yet unknown function for HLA-H. Thus, the principal objective of this study was to describe the main allelic associations between HLA-A and HLA-H, -G, -F and -E. Therefore, HLA-A, -E, -G, -H and -F coding polymorphisms, as well as HLA-G UnTranslated Region haplotypes (referred to as HLA-G UTRs), were explored in 191 voluntary blood donors. Allelic frequencies, Global Linkage Disequilibrium (GLD), Linkage Disequilibrium (LD) for specific pairs of alleles and two-loci haplotype frequencies were estimated. We showed that HLA-A, HLA-H, HLA-F, HLA-G and HLA-G UTRs were all in highly significant pairwise GLD, in contrast to HLA-E. Moreover, HLA-A displayed restricted associations with HLA-G UTR and HLA-H. We also confirmed several associations that were previously found to have a negative impact on transplantation outcome. In summary, our results suggest complex functional and clinical implications of the HLA-A genetic region.

## Introduction

A complete Human Leukocyte Antigen (HLA) match is associated with better long term survival in transplant patients, however the effects of HLA-A, -B and -DRB1 matching are unequal and also depend upon the organ being transplanted. HLA-A mismatches were found to have less influence on kidney allografts than HLA-B mismatches [1]. This difference could reflect the higher number of HLA-B alleles compared to HLA-A and/or the cytotoxic T-cell allo-repertoire for HLA-A and -B antigens, as lower frequencies of Cytotoxic T-Lymphocyte precursor (CTLp) are found for HLA-A mismatches than for HLA-B mismatches [2, 3]. Few amino acid polymorphisms at functionally important positions of the antigen recognition site of the HLA-A molecule have been shown to have a significant influence on clinical outcome [4]. In liver transplant patients however, only HLA-A mismatching was associated to a negative effect on patient survival and a higher recurrence of the hepatitis C virus, whereas no HLA-B and -DR mismatches had no effect [5]. In Lung Transplantation (LTx), the risk of Bronchiolitis Obliterans Syndrome only increased in the presence of two HLA-A mismatches, not for 0 or 1 mismatch or for HLA-B or -DR mismatches [6]. These data support the hypothesis that HLA-A mismatching leads to a particular immunological situation in organ transplantation compared to other loci. The HLA-A molecule displays specific features compared to HLA-B that could account for its particular role: HLA-A and HLA-B alleles carry an unpaired cysteine at different positions of the cytoplasmic tail domain (respectively at positions 339 and 325), which is reported to be involved in recycling, targeting for degradation and influencing recognition by NK receptors as well as in the formation of fully folded MHC class I dimers in exosomes [7]. Other genes, in association/linkage disequilibrium with HLA-A, could also induce either alloreactivity or tolerance. Indeed, the HLA-A locus on chromosome 6 is surrounded by HLA class Ib (non-classical) genes: HLA-E (at the centromeric end), HLA-G, -H and HLA-F (at the telomeric end).

HLA class Ib molecules, HLA-G, HLA-E, HLA-H and HLA-F, are described for their involvement in immune effector cells interactions. They display specific features that differentiate them from HLA class Ia (classical) molecules: a highly conserved peptide binding groove with restricted coding polymorphism and specific expression patterns [8, 9]. Moreover, HLA-G lacks the canonical MHC class I cytoplasmic tail as it is truncated due to the deletion of 6 amino acids. Yet, HLA-G is capable of initiating cellular signaling events upon cross-linking, as HLA-G protein associates with lipid rafts and thus can induce cell proliferation [10, 11].

The immuno-modulating and proliferative properties of HLA class Ib are of great interest in the clinical field, particularly in transplantation. Notably, higher HLA-G expression has been correlated to a better clinical outcome after heart, kidney and Hematopoietic Stem Cell Transplantation (HSCT) (see [12] and [13] for reviews). However, the exploration of HLA-G protein expression is currently limited because micro-environmental expression analysis requires invasive techniques. A further complication is that HLA-G is expressed as many different protein isoforms, generated by alternative splicing, dimer formation, and association with β_2_ microglobulin. Unfortunately, these isoforms are not accurately discriminated by commercial antibodies whereas, most importantly, they are reported to display differential functionality. Primary HLA-G mRNA splicing generates both membrane-bound (HLA-G1 to 4) and soluble (HLA-G5 to 7) isoforms [14] and membrane-bound isoforms can also be shed via proteolytic cleavage by matrix metalloproteinase-2 [15]. The HLA-G1 and -G5 isoforms display a similar structure to HLA class Ia molecules (comprising α1, α2 and α3 domains), whereas HLA-G2 and -G6 are only composed of α1 and α3 domains, -G3 and -G7 contain an α1 domain, and -G4 is composed of α1 and α2 domains [14]. These truncated isoforms appear to be differently processed as HLA-G2, -G3 and -G4 are sensitive to endoglycosidase H, suggesting a non-involvement of the Golgi apparatus, in contrast to HLA-G1 and classical HLA class I [16]. Both HLA-G1 and -G5 isoforms reduce NK cell cytotoxicity with an additive effect [17]. Two studies concluded functional activity for HLA-G2, -G4 and -G6 [16], whereas conflicting results were published concerning HLA-G3 [16, 18]. Besides these differentially spliced isoforms, HLA-G forms disulfide-linked dimers via Cys-42 of the HLA-G α1 domain. Most of the dimers are reported to be expressed on the cell surface, but soluble HLA-G is also able to dimerize [19]. HLA-G dimerization, which may depend on cell-surface density, has an impact on HLA-G receptor affinity [20]. Hence, possibly due to the many HLA-G isoforms and the lack of appropriate serological tools, conflicting results have been reported regarding the association between HLA-G expression and clinical outcome [21–24]. As a consequence, many studies focus on HLA-G genetic polymorphisms to predict clinical outcome.

The many HLA-G regulatory region polymorphisms (5’URR and 3’UTR) are in strong Linkage Disequilibrium (LD) with each other, forming haplotypes referred to as HLA-G UTRs in this manuscript [25]. These HLA-G UTRs are well conserved in different physiological populations, as well as in different clinical cohorts [22–24]. Several clinical studies have described favorable or deleterious effects of HLA-G alleles and HLA-G UTRs. For example, in LTx, HLA-G*01:06~UTR2 was associated with a worse evolution of cystic fibrosis and HLA-G*01:04~UTR3 impaired long term survival [24]. HLA-G*01:06 was associated with pregnancy complications in French [26] and Singaporeans women [27], and an increase of HLA-G*01:04 frequency was observed in couples with recurrent miscarriages [28, 29]. Conversely, HLA-G*01:04 has been shown to be protective in acute renal rejection and end stage renal disease (chronic inflammatory disease) [30].

Another non-classical HLA class I molecule, HLA-E, regulates NK cell function and is the only known ligand for the C-type lectin receptor CD94 combined with different NKG2 sub-units expressed on NK and CD8+ αβ T cells [31, 32]. HLA-E mRNA is expressed in most tissues [33] and its cell surface expression is controlled by leader peptides of HLA class I molecules, except HLA-F, the highest affinity being reported for HLA-G. Similarly to HLA-G, HLA-E’s intrinsic peptide repertoire is constrained by a larger number of primary anchor sites compared to most class Ia molecules, which further supports a specific immunological role for this molecule [34]. HLA-E can also bind peptide ligands from stress proteins and viruses [9, 35]. Although HLA-E possesses less anchor sites than HLA-G, the HLA-E peptide repertoire appears to be more restricted than that of HLA-G. Equal frequencies of the HLA-E coding alleles (HLA-E*01:01 and HLA-E*01:03, R107G, respectively associated to low and high protein expression) suggest that balancing selection may have maintained heterozygosis of high- and low-expressing alleles [36–39]. In HSCT, HLA-E*01:03 homozygosity was associated with a lower risk of Graft Versus Host Disease (GVHD), decreased mortality and a higher disease-free survival rate, whereas HLA-E*01:01 homozygosity was associated with an increased risk of bacterial infection (See [13] for a review). In LTx, HLA-E*01:03 was correlated to Chronic Lung Allograft Dysfunction (CLAD) occurrence and HLA-E homozygosity was associated with a worse survival compared to heterozygosity (Di Cristofaro et al, submitted).

Much less is known about the other HLA class Ib molecules. HLA-F mRNA is expressed in most cell types and the protein is localized in the Endoplasmic Reticulum (ER) and Golgi apparatus. Unlike other HLA class I molecules, the function of HLA-F seems to be independent of peptide loading in the ER [31, 40], as it is exclusively expressed in an open conformer form (OC, deprived of binding peptide and/or β2 microglobulin) since no peptide could be eluted from both membrane-bound and intracellular HLA-F captured proteins [41]. HLA-F is expressed at the surface of the extravillous trophoblast and is upregulated upon activation in monocytes, NK, B and T cells, but not T-Reg CD4+ CD25+ cells [33, 42]. HLA-F seems to possess different functions: interaction with ILT-2 and ILT-4 and with KIR receptors [9, 43]; interaction with HLA-E in the OC form, possibly modifying the interaction with its receptors [44], and an implication in an original pathway of antigen (Ag) cross-presentation by HLA class I molecules [45]. HLA-F is characterized by 4 alleles defined at a 4 digit resolution, with F*01:01 representing 90% of allelic diversity.

Finally, HLA-H has been defined as a pseudogene because of a deletion in exon 4, causing a premature stop codon before cysteine 164, impairing the Ag-presenting function [46]. Of note, HLA-H may be mistaken for the HFE gene (associated with haemochromatosis) [9, 47, 48], but, although the HFE protein is similar to the proteins of the MHC class I family, it does not seem to inhibit NK cell activity [48]. Increased HLA-H nucleotide variation could be the consequence of balancing selection acting on nearby loci or of relaxed functional pressures on this pseudogene [49]. No data is currently available concerning HLA-F or HLA-H alleles and clinical outcome.

Few data exist concerning the associations between HLA class Ib alleles; all located within a region of 724 kb on chromosome 6 (Table 1) [50–52]. So far, the studies on HLA-E reported no LD with other HLA class Ib loci [35], except with HLA-G in isolated populations [53]. However, some HLA-E and HLA-A alleles have been described as being fully associated, e.g. HLA-A*01 and HLA-E*01:01, or HLA-A*23/24/29 and HLA-E*01:03 [36]. HLA-A and HLA-G also display LD [54–56]: HLA-A*23/24 is associated to HLA-G*01:04 and this haplotype displays a deletion of more than 50 kb, comprising HLA-H [51, 57–59]. No data is available concerning HLA-F allelic associations with other HLA class I genes.

**Table 1:** HLA-E, -H, -G, -F and HFE relative location and distance from HLA-A on chromosome 6.

Locus	NCBI Gene ID	Start (bp from pter)	End (bp from pter)	Size (bp)	Distance from HLA-A (Kbp)	Location relative to HLA-A
HLA-E	3133	30489406	30494205	4799	547	Centromeric
HLA-A	3105	29942470	29945884	3414	0	-
HLA-H	3136	29887760	29891080	3320	55	Telomeric
HLA-G	3135	29826979	29831122	4143	115	Telomeric
HLA-F	3134	29887760	29891080	3320	55	Telomeric
HFE	3077	26087281	26096117	8836	3855	Telomeric

Many interactions are described between HLA class Ib molecules and immunological effectors cells, particularly NK cells. Thus, the principal objective of this study is to describe the main allelic associations between the HLA class Ib loci and HLA-A in a healthy population and to address their putative involvement in the synergy of these proteins.

## Materials and Methods

### Clinical samples

Ethylene Diamine Tetra Acetate-anticoagulated (EDTA) peripheral blood samples and sera were collected after written informed consent from 191 voluntary blood donors from southeastern France. The donations were collected in accordance with the French blood donation regulations and ethics and with the French Public Health Code (art L1221-1). The samples were approved by the French Committee for the Protection of Persons in Biomedical Research (CCPPRB) and the entire French collection was also declared to and approved by the French Ministry of Higher Education and Research. Blood samples were anonymized according to the French Blood Center (Etablissement Français du Sang) procedure. Genomic DNA (gDNA) was extracted from a 200-μl total blood sample using the QIAmp Blood DNA Mini kit (Qiagen, Courtaboeuf, France) according to manufacturer’s instructions.

### HLA-A genotyping

Luminex™ technology (HLA-A-One Lambda LABType® SSO, InGen, Chilly Mazarin, France) was used to determine HLA-A alleles at a low resolution level, i. e. 2-digit or first field level typing, using the manufacturer’s kit.

### HLA-G, 5’URR and 3’UTR genotyping

A home-made primer extension method described in [22, 53] was used to simultaneously analyze 16 SNPs of the HLA-G gene: 8 SNPs (codons 13C/T (rs17875397); 31 A/T (rs41551813); 54 A/G (rs41545515); 110 C/A (rs12722477); 130 C/T (rs41557518); 159 T/C (rs55916353); 219 C/T (rs45530733) and 258 C/T (rs12722482)) defining HLA-G*01:01 to G*01:09 alleles, 4 SNPs in the 5’ URR region (-725 C/G/T (rs1233334), -716 G/T (rs2249863), -201 G/A (rs1233333) and -56 C/T (rs17875397)) and 4 SNPs in the 3’UTR region (exon 8 *ins*/*del* (rs66554220); 3142 C/G (rs1063320); 3187 G/A (rs9380142) and 3196 C/G (rs1610696)) defining HLA-G UTR-1 to HLA-G UTR-8. HLA-G genotypes were analyzed using GeneMapper® 4.0 with specific detection parameters.

### HLA-E and HLA-F genotyping

Next Generation Sequencing was used to genotype HLA-E and HLA-F alleles. A fragment of both genes was amplified by PCR with primers designed using the « Primer 3 » program (http://bioinfo.ut.ee/primer3-0.4.0/primer3/). The HLA-E primers flanked the genomic sequence from position 257 to 3072 [2] allowing the typing of E*01:01:01:01/E*01:01:01:02 (non resolved because the ambiguities are at positions -188 and -187, outside the amplified region), E*01:01:01:03, E*01:03:01:01, E*01:03:01:02, E*01:03:02:01, E*01:03:02:02, E*01:03:04 and E*01:06 alleles. The forward and reverse primer sequences were 5’TCATCTCTGTGGGCTACGTG3’ and 5’TCAGACCCCCAGAATCTCAC3’, respectively. The HLA-F primers flanked the genomic sequence from position -266 to 3249 [2] allowing the genotyping of all of the HLA-F alleles described to date. The forward and reverse primer sequences were 5’GTGGCTCTCAAGGGCTCAG3’ and 5’TACGATGCTTTGGTTGTTGC3’, respectively. Both PCR amplifications were performed using the Long Range PCR Kit (Qiagen, Courtaboeuf, France) with 200 ng gDNA in a final volume of 25 μL containing 1x PCR buffer, 0.5 mM of dNTPs, 2 units of Long Range PCR Enzyme Mix and 0.4 μM of each primer. Amplification was carried out as follows: 1 cycle at 95°C for 3 min; 35 cycles at 95°C for 15 sec, 65°C for 30 sec, and 68°C for 5 min. Following PCR amplification, PCR fragments (HLA-E: 2815 bp, HLA-F: 3515 bp) were sequenced using a Next Generation Sequencing platform according to manufacturer’s instructions (MiSeq, Illumina, The Netherlands).

### HLA-H genotyping

Direct sequencing was used to determine HLA-H alleles. A fragment of the HLA-H gene was amplified by PCR with primers designed using the « Primer 3 » program. The primers flanked the genomic sequence from position 284 to 488 [2] allowing the typing of H*01:01:01:01/H*01:01:01:03 (non resolved because the ambiguities are at positions 1252, 1261, 1415, 1427, 1429, 1430 and 1881, outside the amplified region), H*01:01:01:02, H*01:02, H*02:01:01:01, H*02:01:01:02, H*02:02, H*02:03, H*02:04 and H*del alleles. The forward and reverse primer sequences were 5’ ATCTCCGTCGGCTACGTG 3’ and 5’ CGAGTCTCTGGGTCCGAGAT 3’, respectively. PCR amplification was performed on 50 ng of gDNA in a final volume of 25 μL containing 1x PCR buffer, 1.5 mM MgCl2, 0.2 mM of dNTPs, 0.1 unit of Taq DNA-polymerase (Invitrogen, Cergy Pontoise) and 0.16 μM of each primer. Amplification was carried out as follows: 1 cycle at 95°C for 5 min; 30 cycles at 95°C for 30 sec, 57°C for 30 sec, and 72°C for 1 min; and 1 cycle at 72°C for 7 min. PCR amplification was checked by agarose electrophoresis and PCR fragments (length: 293 bp) were sequenced using a Big Dye Terminator V1.1 kit (Life Technologies) with each PCR primer according to the manufacturer’s protocol. Sequences were analyzed using the Codon Code Aligner program (Codon Code Corporation, Massachusetts).

### HLA allelic assignment

The HLA-A, G, H, F and E allelic assignments were based on the HLA sequences listed in the official IMGT/HLA database [2].

HLA-G and HLA-H SNPs were automatically converted from output files (.txt) exported from GeneMapper 4.0 (HLA-G) or from Codon Code Aligner (HLA-H) into alleles and HLA-G UTRs using an in-house computer program readable by the ‘Phenotype’ application of the Gene[Rate] computer tool package (http://hla-net.eu/tools/) [60–62]. The HLA-G interpretation table used by Gene[Rate] is described in [53] and the HLA-H interpretation table is described in S1 Table.

HLA-E and HLA-F genotypes were assigned by the NGSengine program (GenDx, The Netherlands).

For allelic and two or more loci frequency estimations, all HLA-G and HLA-A putative homozygotes were considered either true homozygotes or heterozygotes for both the observed allele and an undefined or undetectable (‘blank’) allele. Due to the deletion encompassing the HLA-H locus, all HLA-H homozygous samples were encoded either as true homozygotes, as heterozygotes for both the observed allele and a ‘blank’ allele or as heterozygotes for both the observed allele and a deleted (‘del’) allele. For HLA-E and HLA-F, this procedure was not applied because typing was performed from NGS data, which allows the identification of any unknown allele (clonal amplification).

### Statistical analysis

Missing data at a locus led to the exclusion of the concerned sample from further analyses at the given locus. No multiple imputations were used.

Allelic and two or more loci haplotype frequencies were estimated using an EM algorithm implemented in the Gene[Rate] computer tools [62]. Deviations from Hardy-Weinberg equilibrium (HWE) were tested using a nested likelihood model [63]. Two-loci Global Linkage Disequilibrium (GLD) was assessed by a likelihood-ratio test on the frequency estimations [60, 61]. LD for specific pairs of alleles was provided as a list of standardized residuals [64] for each observed haplotype and a value greater than |2| was considered to be a significant deviation [60, 61].

## Results

### HLA-A, -E, -H, -F and -G allelic frequencies

HLA-A, -E, -H, -F and -G allele frequencies are given in Table 2. The null hypothesis of Hardy-Weinberg equilibrium was never rejected. Sixteen 2-digit HLA-A alleles were found, and their frequencies were concordant with previously published data in France [61]), HLA-A*02, HLA-A*03, HLA-A*24 and HLA-A*01 being the most frequent alleles. Six 4-digit HLA-G alleles and 8 HLA-G UTRs were observed and their frequencies were similar to previously published data [22, 53]. Putative undefined or undetectable blank ‘alleles’ represented at the most 1.6% of HLA-G UTRs according to the EM estimation (Table 2). Seven 8-digit HLA-E alleles were found; their frequencies were concordant with published data showing an equal distribution between HLA-E*01:01 and HLA-E*01:03, with HLA-E*01:03:02:01 being the most frequent allele in Western Europe [53, 65]. The HLA-E*01:03:01:02 and HLA-E*01:03:04 alleles were not observed, while HLA-E*01:06 and HLA-E*01:03:02:02 displayed very low frequencies (<0.5%). Eight 6-digit HLA-H alleles were found, HLA-H*01:01:01 and HLA-H*02:04 being the most frequent. A new HLA-H allele (H*02:04new) was identified at a frequency of 8.7% in this study, defined by a silent G>A substitution at position 368 compared to the IMGT gDNA reference for H*02:04 (which was observed at a frequency of 15.9%). The HLA-H*del was found at a frequency of 13.9%. HLA-H*01:01:01:02, H*01:01:01:03 and H*02:01:01:02 were not observed and are further referred to as H*01:01:01 and H*02:01:01, respectively. Finally, four 6-digit HLA-F alleles were found, F*01:01:01 being the most frequent while HLA-F*01:02 and F*01:04 were not observed.

**Table 2:** HLA-A, HLA-G UTRs, -G, -E, -H and -F allelic frequencies in 191 voluntary blood donors from southeastern France.

Allele	Fq (%)	Allele	Fq (%)
A*02	31.6	G*01:01	78.3
A*03	15.0	G*01:04	11.5
A*24	10.8	G*01:06	3.9
A*01	10.3	G*01:03	3.9
A*11	7.1	G*01:05N	2.1
A*29	6.6	G*01:08	0.3
A*30	3.7	E*01:01:01:01	47.5
A*31	2.6	E*01:03:02:01	42.1
A*26	2.6	E*01:03:01:01	5.0
A*23	2.4	E*01:01:01:03	2.5
A*68	2.1	E*01:03:05	2.0
A*33	1.8	E*01:06	0.5
A*32	1.8	E*01:03:02:02	0.5
A*25	1.3	H*01:01:01	33.9
A*80	0.3	H*02:04	15.9
HLA-G UTR1	31.3	H*02:01:01	15.1
HLA-G UTR2	21.8	H*del	13.9
HLA-G UTR4	14.0	H*02:04new	8.7
HLA-G UTR3	11.7	H*02:02	6.0
HLA-G UTR6	7.5	H*01:02	3.8
HLA-G UTR7	7.1	H*02:03	2.7
HLA-G UTR5	4.8	F*01:01:01	46.2
Blank	1.6	F*01:01:03	21.8
HLA-G UTR8	0.3	F*01:03:01	18.6
		F*01:01:02	13.5

### Two loci linkage disequilibrium and haplotypes

Analysis of two-loci Global Linkage Disequilibrium (GLD) (Table 3) showed that HLA-A, HLA-H, HLA-F, HLA-G and HLA-G UTRs were all in highly significant pairwise GLD (p<0.001). In contrast, the HLA-E locus was not in significant GLD with any of them. However, when defined at a 4-digit resolution level, HLA-E displayed a significant GLD with HLA-F but the p-value of 1% was not significant after adjusting for multiple testing (Table 3). Haplotypes were estimated between each pair of loci and their combined frequencies are given in Table 4. Strikingly, a strong LD was found between HLA-A, HLA-G UTRs and HLA-H, as all haplotypes with a frequency above 3% and concerning these pairs of loci were in significant LD. These loci were also found to be highly associated with HLA-F, as most haplotypes with a frequency above 3% were also in LD.

**Table 3:** Analysis of two-loci global linkage disequilibrium (GLD) between HLA-E (defined at 8 (8d) or 4-digits (4d)) and HLA-A, -H, -G, HLA-G UTRs and -F.

HLA-E 8d	HLA-E 4d					
0.81	0.39	HLA-A				
0.34	0.08	<0.001	HLA-H			
0.99	0.85	<0.001	<0.001	HLA-G		
0.62	0.25	<0.001	<0.001	<0.001	HLA-G UTRs	
0.09	0.01 *	<0.001	<0.001	<0.001	<0.001	HLA-F

* non-significant after adjusting for multiple testing.

**Table 4:** Number (N) of haplotypes observed at frequencies (fq) greater or equal to 3% and 1% and their combined frequencies (F_C_) in 191 voluntary blood donors from southeastern France.

Pairs of loci	N of haplotypes with a fq≥1% (and Fc)	N of haplotypes with a fq≥3% (and Fc)	N of haplotypes with a fq≥3% in LD
HLA-A~ HLA-G UTR	18 (94.9)	6 (74.8)	6
HLA-A~HLA-H	19 (94.9)	6 (73.3)	6
HLA-A~HLA-F	22 (97.1)	9 (76.1)	6
HLA-A~HLA-G	19 (97.4)	7 (77.1)	2
HLA-A~HLA-E	27 (96.4)	9 (71.4)	3
HLA-H~HLA-G UTR	13 (93.8)	8 (85.3)	8
HLA-F~HLA-G UTR	13 (98.3)	9 (89.2)	6
HLA-E~HLA-G UTR	18 (96.5)	10 (82.4)	4
HLA-G~HLA-G UTR	10 (98.5)	8 (95.3)	4
HLA-H~HLA-F	18 (97.2)	8 (79.1)	5
HLA-H~HLA-G	12 (98.3)	9 (91.4)	3
HLA-E~HLA-H	16 (95.1)	8 (80.1)	2
HLA-E~HLA-F	12 (98.1)	7 (90.3)	2
HLA-E~HLA-G	10 (94.5)	6 (86.3)	0

The detailed description of the haplotypes observed with a frequency greater than 3% (or 2% for those involving HLA-A; Table 5) reveals significant allelic associations between HLA-A, HLA-G UTRs and HLA-H, respectively, with very high standardized residual values. HLA-F also displays significant allelic associations with HLA-A, HLA-G UTRs and HLA-H, but to a lower extent. In contrast, HLA-G alleles display few significant associations with HLA-A, HLA-H, HLA-F and HLA-G UTRs, with the exception of HLA-G*01:03, *01:04 and *01:06. Finally, HLA-E reveals some significant associations, but the standardized residuals are very close to the threshold value for significance.

**Table 5:** Two-loci haplotypes with a frequency higher than 3% (2% for HLA-A); obs: estimated frequency; exp: expected frequency; stdres: standardized residual–in grey if significant, i.e. corresponding to an absolute value above 2.

Loci	obs	exp	stdres												
A*2~UTR1	28.1	9.8	10.62												
A*1~UTR2	10.5	2.4	10.11												
A*3~UTR4	13.8	2.2	15.00												
A*24~UTR3	8.9	1.3	12.82												
A*23~UTR3	2.4	0.3	7.76												
A*11~UTR7	7.0	0.5	17.45												
A*29~UTR6	6.5	0.5	16.03												
A*26~UTR2	2.4	0.5	4.90												
Loci	obs	exp	stdres	Loci	obs	exp	stdres								
A*2~H*01:01:01	30.5	10.8	10.92	UTR1~H*01:01:01	29.0	10.4	10.41								
A*1~H*02:01:01	9.9	1.6	12.52	UTR2~H*02:01:01	14.0	3.4	10.81								
A*3~H*02:04	11.4	2.3	11.32	UTR4~H*02:04	11.7	2.2	12.30								
A*24~H*del	8.5	1.3	11.81	UTR3~H*del	11.5	1.6	14.72								
A*11~H*02:04new	7.0	0.6	15.53	UTR7~H*02:04new	6.9	0.6	15.24								
A*29~H*02:02	6.0	0.4	16.63	UTR6~H*02:02	5.8	0.5	14.67								
A*31~H*02:04:01	2.4	0.4	5.83	UTR2~H*01:02	3.2	0.9	4.71								
A*26~H*01:02	2.4	0.1	14.10	UTR5~H*02:04	3.3	0.7	5.71								
Loci	obs	exp	stdres	Loci	obs	exp	stdres	Loci	obs	exp	stdres				
A*2~F*01:01:01	26.1	13.2	4.13	UTR1~F*01:01:01	29.3	13.6	4.86	H*01:01:01~F*01:01:01	27.3	14.3	3.94				
A*3~F*01:03:01	13.0	2.9	7.18	UTR4~F*01:03:01	14.7	2.8	8.48	H*02:04~F*01:03:01	13.8	3.6	6.52				
A*1~F*01:01:03	9.7	2.1	6.50	UTR2~F*01:01:03	13.4	4.8	4.67	H*02:01:01~F*01:01:03	12.3	3.3	5.99				
A*11~F*01:01:02	6.5	1.1	6.45	UTR6~F*01:01:01	6.5	3.3	2.10	del~F*01:01:03	6.2	2.6	2.70				
A*29~F*01:01:01	5.7	3.3	1.63	UTR7~F*01:01:02	5.9	1.0	6.09	H*02:04new~F*01:01:02	5.8	1.2	5.19				
A*24~F*01:01:01	4.0	4.2	-0.10	UTR2~F*01:01:01	5.7	10.0	-1.56	H*02:02~F*01:01:01	5.1	3.1	1.40				
A*24~F*01:01:03	4.0	1.9	1.81	UTR3~F*01:01:03	5.0	2.6	1.74	del~F*01:01:01	4.7	5.5	-0.41				
A*31~F*01:01:03	3.8	1.0	3.52	UTR3~F*01:03:01	4.7	2.3	1.87	H*01:02~F*01:01:01	3.9	1.8	1.93				
A*23~F*01:03:01	3.3	0.6	4.17	UTR5~F*01:01:02	4.0	0.9	4.02								
A*33~F*01:01:02	2.6	0.4	4.67												
A*26~F*01:01:01	2.6	1.2	1.58												
Loci	obs	exp	stdres	Loci	obs	exp	stdres	Loci	obs	exp	stdres	Loci	obs	exp	stdres
A*2~G*01:01	30.3	24.8	1.87	UTR1~G*01:01	31.4	24.3	2.42	H*01:01:01~G*01:01	31.2	26.6	1.44	F*01:01:01~G*01:01	45.0	35.2	1.66
A*3~G*01:01	14.2	11.8	1.31	UTR2~G*01:01	15.6	17.0	-0.58	H*02:04~G*01:01	13.1	12.5	0.30	F*01:03:01~G*01:01	15.3	14.2	0.35
A*24~G*01:04	8.7	1.2	13.06	UTR4~G*01:01	14.1	10.9	1.77	H*del~G*01:04	11.3	1.6	14.86	F*01:01:02~G*01:01	8.8	10.3	-0.55
A*11~G*01:01	7.1	5.6	1.23	UTR3~G*01:04	11.8	1.4	17.05	H*02:01:01~G*01:01	9.0	11.8	-1.47	F*01:01:03~G*01:01	7.2	16.6	-2.64
A*29~G*01:01	6.6	5.2	1.19	UTR7~G*01:01	7.2	5.6	1.30	H*02:04new~G*01:01	8.7	6.8	1.35	F*01:01:03~G*01:04	6.0	2.5	2.72
A*1~G*01:01	6.3	8.0	-1.14	UTR6~G*01:01	7.5	5.8	1.29	H*02:02~G*01:01	5.9	4.7	1.00	F*01:01:03~G*01:06	4.5	1.0	4.41
A*1~G*01:06	4.0	0.4	10.83	UTR2~G*01:06	4.0	0.9	6.46	H*del~G*01:01	4.4	10.9	-3.59	F*01:01:02~G*01:03	3.6	0.7	4.33
A*26~G*01:01	2.6	2.1	0.76	UTR5~G*01:03	3.7	0.2	15.60	H*02:01:01~G*01:06	4.0	0.6	8.45	F*01:03:01~G*01:04	3.3	2.1	0.94
A*23~G*01:04	2.4	0.3	7.90					H*01:02~G*01:01	3.9	3.0	0.92				
A*24~G*01:01	2.1	8.5	-4.07												
A*30~G*01:01	2.1	2.9	-0.92												
Loci	obs	exp	stdres	Loci	obs	exp	stdres	Loci	obs	exp	stdres	Loci	obs	exp	stdres
A*2~E*01:03:02:01	15.2	12.1	1.18	E*01:01:01:01~UTR2	16.9	11.0	2.34	E*01:03:02:01~H*01:01:01	17.3	13.0	1.59	F*01:01:01~E*01:03:02:01	25.8	19.8	1.49
A*2~E*01:01:01:01	11.1	13.5	-0.87	E*01:03:02:01~UTR1	14.1	12.2	0.69	E*01:01:01:01~H*02:01:01	13.5	7.4	3.03	F*01:01:03~E*01:01:01:01	17.0	10.3	2.47
A*3~E*01:03:02:01	10.4	6.6	2.05	E*01:01:01:01~UTR1	12.3	13.4	-0.41	E*01:01:01:01~H*01:01:01	10.6	14.6	-1.40	F*01:01:01~E*01:01:01:01	16.4	21.9	-1.31
A*1~E*01:01:01:01	8.9	4.5	2.87	E*01:03:02:01~UTR4	11.2	6.1	2.76	E*01:03:02:01~H*02:04	10.3	8.5	0.83	F*01:03:01~E*01:03:02:01	13.7	8.0	2.42
A*29~E*01:03:02:01	8.5	3.6	3.57	E*01:03:02:01~UTR6	7.7	3.9	2.60	E*01:01:01:01~H*02:04	9.4	9.6	-0.09	F*01:01:02~E*01:01:01:01	9.8	6.4	1.64
A*3~E*01:01:01:01	5.1	7.4	-1.15	E*01:01:01:01~UTR3	5.4	5.3	0.09	E*01:03:02:01~H*02:02	8.0	3.5	3.37	F*01:03:01~E*01:01:01:01	4.2	8.8	-1.84
A*11~E*01:01:01:01	4.6	3.3	0.97	E*01:01:01:01~UTR7	4.4	3.1	1.01	E*01:01:01:01~H*del	7.3	5.3	1.17	F*01:01:01~E*01:03:01:01	3.4	2.1	1.14
A*24~E*01:01:01:01	4.5	4.3	0.18	E*01:03:02:01~UTR2	4.0	10.1	-2.53	E*01:01:01:01~H*02:04new	4.3	3.5	0.60				
A*33~E*01:01:01:01	3.0	1.4	1.85	E*01:01:01:01~UTR5	3.3	3.1	0.17					Loci	obs	exp	stdres
A*24~E*01:03:02:01	2.2	3.8	-1.13	E*01:01:01:01~UTR4	3.1	6.7	-1.87					F*01:01:01~E*0103	32.4	23.4	2.05
A*23~E*01:01:01:01	2.0	1.4	0.68									F*01:01:03~E*0101	18.6	10.6	2.90
A*31~E*01:03:02:01	2.0	1.9	0.09									F*01:01:01~E*0101	13.7	22.5	-2.03
A*32~E*01:01:01:01	2.0	1.0	1.52									F*01:03:01~E*0103	13.2	9.4	1.48
												F*01:01:02~E*0101	11.0	6.6	2.10
												F*01:03:01~E*0101	5.4	9.1	-1.47
												F*01:01:03~E*0103	3.2	11.0	-2.79

The tight association found between HLA-A, HLA-G UTRs and HLA-H is further supported by the three-loci haplotype frequency estimation shown in Table 6. HLA-G UTR3 is associated with HLA-H*del and HLA-A*23/24 alleles, HLA-G UTR6 is associated with HLA-H*02:02 and HLA-A*29, and HLA-G UTR7 is associated with HLA-H*02:04new and HLA-A*11, whereas HLA-G UTR2 displays no exclusive association.

**Table 6:** HLA-A~HLA-H~ HLA-G UTRs haplotypes with a frequency greater than 1%.

Haplotypes	Frequency
A*01~H*02:01:01~ HLA-G UTR2	10.21
A*02~H*01:01:01~HLA-G UTR 1	27.53
A*02~H*01:01:01~HLA-G UTR 5	1.39
A*02~H*01:01:01~HLA-G UTR 2	1.12
A*03~H*02:04~ HLA-G UTR 4	11.6
A*03~H*02:04new~HLA-G UTR 4	1.67
A*11~H*02:04new~HLA-G UTR 7	6.92
A*23~H*null~HLA-G UTR 3	1.11
A*23~H*del~HLA-G UTR 3	1.11
A*24~H*del~HLA-G UTR3	8.78
A*25~H*01:02~HLA-G UTR2	1.11
A*26~H*01:02~HLA-G UTR2	2.22
A*29~H*02:02~HLA-G UTR6	5.83
A*30~H*01:01:01~HLA-G UTR1	1.67
A*30~H*02:01:01~HLA-G UTR2	1.39
A*31~H*02:04~HLA-G UTR5	1.39
A*33~H*02:04~HLA-G UTR5	1.94
A*68~H*02:01:01~HLA-G UTR2	1.65

## Discussion

This study was devoted to the analysis of allelic associations between the HLA class Ia locus HLA-A, and the HLA class Ib loci HLA-E, -H, -G, HLA-G UTRs and -F which are all physically located within a region of 724 kb on chromosome 6. HLA-A seems to be involved in transplantation outcome in a different way from HLA-B or HLA-DRB1 [1, 3]. This singular feature could be due to the HLA-A molecule *per se* and/or be a consequence of yet unknown patterns of linkage disequilibrium between HLA-A and nearby class Ib genes.

Currently, interactions between HLA class Ib molecules are barely known, and even less so in the clinical setting. Genetic phylogeny is one approach that might shed some light on this subject, as it has been suggested that 1) HLA-A and -H share a more recent ancestor than HLA-A with either HLA-B or C and that 2) HLA-E is more closely related to HLA-H than to -F or -G [46]. Moreover, HLA-E alleles are thought to pre-date most of the HLA-A and HLA-B polymorphisms [36]. However, an evolutionary perspective on class Ib polymorphisms would probably not be very informative regarding clinical implications.

Another possible approach to tackle this question was adopted here by looking at patterns of association previously described to have a clinical impact. Furthermore we have added to the interest of analyzing HLA-G UTRs in clinical studies as some of them display very specific associations, such as HLA-G UTR-4 associated with HLA-A*03, HLA-H*02:04 and HLA-F*01:03; HLA-G UTR-6 associated with HLA-A*29, HLA-H*02:02 and HLA-F*01:01; and HLA-G UTR-7 associated with HLA-A*11, HLA-H*02:04new and HLA-F*01:01. In turn, HLA-G UTR-2 certainly needs further investigation as it was observed in association with several HLA-A, -H and -F alleles, probably consisting of at least 2 clusters with respect to HLA-H and HLA-G.

Of interest, several studies have described a deleterious association of HLA-G*01:06~UTR2 and HLA-G*01:04~UTR3, in lung transplantation and pregnancy [24, 26–29]. In this study we confirmed the exclusive association of HLA-G*01:04 with HLA-G UTR3, HLA-A*23/*24 and the deletion encompassing HLA-H. HLA-E and HLA-F did not display an exclusive association with HLA-G*01:04, as this allele was associated with both F*01:01:03 (6%) and F*01:03:01 (3.3%), and with E*01:01:01 (5.4%) and E*01:03 (combined frequencies: 5.7%). Thus, we did not observe an exclusive association between HLA A*23/*24 and E*01:01 [36] could not be confirmed here. Whether the worse LTx progression reported for the HLA-G*01:04 allele is due to HLA-A*23/24 or to the deletion (encompassing HCG4P5, HLA-U, HLA-K, HCG4B, DDX39BP, MCCD1P1, HLA-T, HLA-H, HCG4P7, MICF and HCG3-2) remains to be explored. Noteworthy, this deletion, always associated to HLA-A*23 and A*24, was also found in HLA-G*01:01 carriers (4.4%). Interestingly, the HLA-H locus, translated into a truncated protein, has a signal sequence similar to that of HLA-A, except that the second position (Valine) is identical to HLA-G and the third position displays a specific amino-acid (Val>Leu) compared to other HLA class I proteins (Table 7) [2]. Interestingly, the amino-acid in the 10^th^ position is identical to HLA-A (Leu) and different from HLA-G (Phe). This characteristic of the HLA-G peptide signal is put forward to explain a higher affinity of the HLA-G-derived nonamer-HLA-E complex with CD94/NKG2C receptor complex [66]. Whether the HLA-H signal sequence is loaded by HLA-E and preferentially protects cells from NK lysis remains to be explored, but the absence of HLA-H could potentially have an impact on the interaction of HLA-E with its receptors.

**Table 7:** HLA-H, HLA-A and HLA-G signal sequence alignment. Variable amino-acid positions among loci are highlighted in bold.

Locus	Signal sequence
HLA-A	M**A**VMAPRTLLLLLSGALALTQTWA
HLA-G	MVVMAPRTL**F**LLLSGAL**T**LT**E**TWA
HLA-H	MV**L**MAPRTLLLLLSGALALTQTWA

Concerning HLA-G*01:06, it displayed a restricted association pattern as it was associated with HLA-G UTR-2, HLA-A*01, HLA-H*02:01:01 and HLA-F*01:01:03, and in positive LD with HLA-E*01:01:01 and negative LD with HLA-E*01:03:02. Thus, the negative clinical effect of HLA-G*01:06 could be the combined result of lower immune-tolerance due to reduced HLA-G expression expected from the association with HLA-G UTR-2 [23] and to reduced HLA-E expression expected from the association with HLA-E*01:01 [35]; and yet unknown features of HLA-H*02:01:01 and HLA-F*01:01:03. Confirming such a hypothesis demands a much larger cohort, because of the low allelic frequency of HLA-G*01:06 (3.9%), and would also greatly benefit from functional studies on HLA-H and HLA-F.

Looking now at the peculiar pattern of linkage disequilibrium revealed by HLA-E, its large physical distance from other HLA class Ib loci (>660Kb), as well as balancing selective forces acting on this locus could be put forward to explain the absence of GLD. However, the functional and clinical implications of HLA-E alleles not being associated to other HLA class Ib loci remain to be formally investigated.

In an HLA class Ia matching transplant context, however, the significant association observed between E*01:01 and A*01 on the one hand, and between E*01:03 and A*29, on the other hand, as shown in [36], implies that no HLA-E heterozygosity should be expected for most HLA-A*01 and A*29 carriers.

Finally, besides protein modifications and differential levels of expression that could impact their respective interaction and affinity with their specific receptors, non-classical HLA polymorphisms could also influence antibody production. Non-native forms of HLA-Ib, caused by alternative splicing or loss of β-2 microglobulin, may expose immunogenic cryptic epitopes and trigger an immune response [67]. Naturally occurring anti-HLA-E antibodies were shown to mimic anti-HLA-Ia antibodies and such cross-reactive binding was reported to affect the screening of anti-HLA-Ia antibodies in renal and liver transplant recipients [68]. Anti-HLA-F IgG, associated with the inflammatory status of systemic lupus erythematosus, may represent a biomarker of primary importance in transplantation, although their exact function has not yet been fully elucidated [69].

In conclusion, this study offers a unique view of non-classical HLA class I allelic associations that may be helpful for future functional studies in deciphering HLA class Ib protein interactions. Since HLA-A typing of recipients and donors is systematically and routinely performed in immunogenetics laboratories, these results could also potentially improve transplant matching strategies.

## Supporting Information

S1 Table Allelic conversion table for HLA-H SNPs. (DOC)

## Abbreviations

HLA: Human Leukocyte Antigen
CTL: Cytotoxic T-Lymphocyte
NK: Natural Killer cells
MHC: Major Histocompatibility Complex
LD: Linkage Disequilibrium
ILT: Immunoglobulin-Like Transcript
KIR: Killer-cell Immunoglobulin-like Receptor
ER: Endoplasmic Reticulum
OC: Open Conformer
HSCT: Hematopoietic Stem Cell Transplantation
β2m: β2-microglobulin
UTR: UnTranslated Region
URR: Upstream Regulatory Region
LTX: Lung Transplantation
GVHD: Graft Versus Host Disease
CLAD: Chronic Lung Allograft Dysfunction
gDNA: genomic DNA
