# Supplementary material for: Association of HLA-A and Non-Classical HLA Class I Alleles
Source: PLoS One. 2016 Oct 4;11(10):e0163570. doi: 10.1371/journal.pone.0163570 (PMC5049754; doi:10.1371/journal.pone.0163570)
Supplement: S1 Table — (DOC) [file pone.0163570.s001.doc]

**Supplementary S1 Table** Allelic conversion table for HLA-H SNPs

284_G=H*01:01:01:01:H*01:01:01:02:H*01:01:01:03:H*01:02:H*02:01:01:01:H*02:01:01:02:H*02:03:H*02:04:H*02:04new

284_T=H*02:02

315_C=H*01:01:01:01:H*01:01:01:02:H*01:01:01:03:H*01:02

315_A=H*02:01:01:01:H*02:01:01:02:H*02:02:H*02:03:H*02:04:H*02:04new

322_C=H*01:01:01:01:H*01:01:01:02:H*01:01:01:03:H*01:02

322_T=H*02:01:01:01:H*02:01:01:02:H*02:02:H*02:03:H*02:04:H*02:04new

324_A=H*01:01:01:01:H*01:01:01:02:H*01:01:01:03:H*01:02

324_C=H*02:01:01:01:H*02:01:01:02:H*02:02:H*02:03:H*02:04:H*02:04new

328_G=H*01:01:01:01:H*01:01:01:02:H*01:01:01:03:H*01:02

328_A=H*02:01:01:01:H*02:01:01:02:H*02:02:H*02:03:H*02:04:H*02:04new

329_A=H*01:01:01:01:H*01:01:01:02:H*01:01:01:03:H*01:02

329_G=H*02:01:01:01:H*02:01:01:02:H*02:02:H*02:03:H*02:04:H*02:04new

330_T=H*01:01:01:01:H*01:01:01:02:H*01:01:01:03:H*01:02

330_A=H*02:01:01:01:H*02:01:01:02:H*02:02:H*02:03:H*02:04:H*02:04new

367_G=H*01:01:01:01:H*01:01:01:02:H*01:01:01:03:H*01:02:H*02:01:01:01:H*02:01:01:02:H*02:02:H*02:03

367_A=H*02:04:H*02:04new

368_G=H*01:01:01:01:H*01:01:01:02:H*01:01:01:03:H*01:02:H*02:01:01:01:H*02:01:01:02:H*02:02:H*02:03:H*02:04

368_A=H*02:04new

406_G=H*01:01:01:01:H*01:01:01:02:H*01:01:01:03:H*01:02:H*02:04:H*02:04new

406_A=H*02:01:01:01:H*02:01:01:02:H*02:02:H*02:03

411_A=H*01:01:01:01:H*01:01:01:02:H*01:01:01:03:H*01:02:H*02:04:H*02:04new

411_G=H*02:01:01:01:H*02:01:01:02:H*02:02:H*02:03

444_G=H*01:01:01:01:H*01:01:01:02:H*01:01:01:03:H*02:01:01:01:H*02:01:01:02:H*02:02:H*02:03:H*02:04:H*02:04new

444_T=H*01:02

468_G=H*01:01:01:01:H*01:01:01:02:H*01:01:01:03:H*01:02:H*02:01:01:01:H*02:02:H*02:03:H*02:04:H*02:04new

468_T=H*02:01:01:02

487_G=H*01:01:01:01:H*01:01:01:03:H*01:02:H*02:01:01:01:H*02:01:01:02:H*02:02:H*02:03:H*02:04:H*02:04new

487_A=H*01:01:01:02

488_A=H*01:01:01:01:H*01:01:01:02:H*01:01:01:03:H*01:02:H*02:02:H*02:03

488_G=H*02:01:01:01:H*02:01:01:02:H*02:04:H*02:04new
